# Psychosocial Predictors of Cognitive Impairment in the Elderly: A Cross-Sectional Study 

**Published:** 2018-07

**Authors:** Mahbobeh Faramarzi, Maryam Zarin Kamar, Farzan Kheirkhah, Ahmad Karkhah, Ali Bijani, Seyed Reza Hosseini

**Affiliations:** 1 Social Determinants of Health (SDH) Research Centre, Health Research Institute, Babol University of Medicine Sciences, Babol, Iran.; 2 Student Research Committee, School of Medicine, Babol University of Medicine Sciences, Babol, Iran.; 3 Neuroscience Research Center, Health Research Institute, Babol University of Medicine Sciences, Babol, Iran.; 4 Cellular and Molecular Biology Research Center, Student Research Committee, School of Medicine, Babol University of Medicine Sciences, Babol, Iran.

**Keywords:** *Cognitive Impairment*, *Depressive Symptoms*, *Older People*, *Predictors*, *Social Support*

## Abstract

**Objective:** Cognitive impairment is a major public health problem among elderly population. The aim of this study was to assess some psychosocial predictors of cognitive impairment (age, education, living alone, smoking, depression and social support) in the Iranian elderly population.

**Method** **:** A total of 1612 elderly (over 60 years) were enrolled in this cross-sectional study. Cognitive function was assessed using Mini Mental State Examination (MMSE). In addition, data from psychological tests and demographic characteristics were analyzed.

**Results: **Older age, low education level, living alone, smoking, depressive symptoms, and lower social support were associated with an increased risk of cognitive impairment. Ages 70 to 74 (OR = 3.47; 95% CI, 2.13-5.65), 75 to79 (OR = 3.05; 95% CI, 2.11-4.41) and 80 to 85 (OR = 5.81; 95% CI, 2.99-11.22) and depression symptoms (OR = 1.64; 95% CI, 1.27-2.13) were significant positive predictors, whereas social support with scores ranging from 26 to 30 (OR =0. 32; 95% CI, 0.16-0.62) and 31 to 33 (OR =0.29; 95% CI, 0.14-0.61) and more than 5 years of education (OR = 0.19; 95% CI, 0.14-0.27) were the negative predictors of cognitive impairment.

**Conclusion: **The findings suggest older age and depression as positive predictive factors and higher education level and social support as negative predictive factors of cognitive impairment in the elderly population.

Dementia is the common cause of cognitive impairment in the elderly population. Dementia reflects disruption in one or more cognition domains (memory, language, orientation, judgment, conducting interpersonal relationship, performing actions, and problem solving) and is frequently complicated by behavioural symptoms (1). Dementia is a common illness, which is known as the most important cause of disability and mortality in older adults (2). According to World Health Organization (WHO) reports, the prevalence of dementia ranges from 2.1% to 8.1% in the older population aged 60 years and over (3). 

Alzheimer’s disease, vascular dementia, dementia with Lewy bodies, and frontotemporal dementia are the most common types of dementia (4). Some studies have introduced several risk factors for dementia (5-8). Beerenset et al. suggested that depressive symptoms and agitation are related to lower quality of life in the elderly with dementia (5). In addition, Wei et al. showed that stroke, lack of fruits and vegetables in daily diet, early parental death, household ﬁnancial management, cardiovascular disease, and hypertension were signiﬁcantly associated with dementia in highly educated elderly people in Tianjin (6). 

Another study found that 75% of patients suffering from dementia have experienced some neuropsychiatric symptoms in the past month (apathy 36%, depression 32%, agitation 30%) and that 80% of patients experienced at least one symptom at the onset of cognitive decline (7). 

Depression is one of the most prevalent symptoms in dementia. It is not well-known whether depression could lead to cognitive impairment (10). Some meta-analysis studies found that individuals with a history of depression were more predisposed to Alzheimer’s dementia during ageing (11, 12). Additionally, some investigations suggest that depression is a psychological reaction to cognitive decline (13, 14).

Social support has a strong influence on older individuals’ health (15). Social support is a general concept commonly divided into instrumental and emotional support. Social support refers to the number of social relationships of individuals and its quality (16). Several lines of evidence emphasize that higher cognitive function in older adults was associated with increased social support (17). Taken together, identifying factors of successful cognitive aging influences prevention strategies to ensure optimal health and prevent or delay cognitive impairment in the elderly. Although previous literature investigated the risk factors of cognitive impairment, few studies have focused on the psychological risk factors (10-14). In this cross-sectional study, we assessed some psychosocial predictive factors of dementia (age, education, living alone, smoking, depression, social support) in the Iranian elderly.

## Materials and Methods

***Study Population and Design***

This cross-sectional study was a part of the Amirkola Health and Ageing Project (AHAP) (18). AHAP cohort study is mainly concerned with geriatric medical problems, such as falling, bone fragility and fractures, cognitive impairment and dementia, poor mobility, and functional dependence. Those aged 60 years and over were invited to participate in this study through posters distributed throughout the city and were assessed by a broad range of biochemical and hormonal tests measured at baseline and follow-up. All participants signed a written informed consent. AHAP cohort study was approved by the Medical Ethics Committee of Babol University of Medical Sciences. A detailed discussion of the AHAP sample was described previously (18). 

Health care practitioners were trained in a workshop to use the questionnaire and assess the old population in the public health clinics of city of Amirkola. Inclusion criteria were as follow: age 60 and over and completing all the 3 questionnaires of Mini Mental State Examination (MMSE), Duke Social Support Index (DSSI), and Geriatric Depression Scale (GDS). Elderly people who were not able to answer questions due to severe psychiatric disorders, such as bipolar mood disorder, depressive disorder or psychosis, were excluded.

**Assessment of Cognition**

***Mini Mental State Examination (MMSE)***

MMSE is a brief and objective screening tool for cognitive impairment among the elderly that has proven to be valid and reliable across a variety of clinical, epidemiological, and community survey studies. It is used to evaluate various aspects of cognitive function, including orientation, attention, memory, language, and visual-spatial skills (19). The total score for the MMSE ranges from 0 to 30. The most widely accepted and frequently used cut- off score for the MMSE is 25, with the score 25 or lower indicating the presence of cognitive impairment. Because the MMSE was developed as a screening tool for cognitive impairment, a low score (≤ 24) indicates both the likelihood of cognitive impairment and the need for further evaluation. In this study, we considered MMSE score of 25 to 30 as normal, 20 to 24 as mild cognitive impairment, 10 to 19 as moderate cognitive impairment, and ≤ 9 as severe cognitive impairment (20, 21). Also, the scores less than 24 were adjusted based on age and education level using the score adjustment coefficients suggested by Magni et al. (22). 

***Duke Social Support Index (DSSI)***

This instrument consists of 11 questions and 2 subscales: social interaction (4 items) and social satisfaction (7 items). The items were scored using a Likert scale as follow: 1 point for rarely/very dissatisfied, 2 points for sometimes/dissatisfied, and 3 points for most of the times /satisfied. Total social support scores range from 11 to 33. A higher score indicates higher levels of social support. Internal consistency for all the questions was 0.77, and test-retest reliability was 0.70 to 0.81 (23). In addition, we used the validated Persian version of DSSI (24). The Cronbach's alpha of the scale was 0.69 in this study.

***Geriatric Depression Scale (GDS)***

 This instrument is a valid questionnaire (25), with 15 questions, each of which is scored 0 or 1. Ten questions suggest the presence of depression when answered positively, while the rest (question numbers 1, 5, 7, 11, 13) suggest depression when answered negatively. A low score (≤ 24) indicates depressive symptoms, which were divided into mild (5-8), moderate (9-12), and severe (13-15). Test sensitivity and specificity were 92% and 89%, respectively (25-27). In addition, we used the validated Persian GDS to assess depression (28). Cronbach's alpha for this questionnaire was 0.81 in the elderly population of Amirkola city.

**Data Analysis**

At first, descriptive analysis was conducted to compute the percentages, means, and standard deviations of all variables. T tests were used to identify the significant differences between men and women. Chi- square tests were used to identify univariate relationships between the psychosocial variables and cognition impairment. At first, each single variable was entered into the univariate logistic regression. Then, condition variables (P<0.20) were entered into the final multivariate model. Backward multivariate logistic regression analysis was performed to assess independent predictors of cognition impairment and odds ratios (OR), and 95% CI were estimated. This model was then adjusted for all baseline characteristics that were significant at p <0.05 in the bivariate analysis. All analyses were conducted using SPSS software Version 18.

## Results

A total of 1616 older people participated in this study (72.3% response rate), and 4 patients were excluded due to incompletion the evaluation for cognitive impairment. Therefore, 1612 participants were entered in this study. Among them, 881 (54.7%) were male and 731 (45.3%) were female. Table 1 demonstrates the demographic characteristics of the participants.

In this study, the mean social support score was 27.48 ± 2.98. According to GDS scores, 700 (43.4%) individuals had symptoms of depression (mild 63%, moderate 25.2%, and severe 11.8%). Based on MMSE scores, 509 (31.5%) individuals had cognitive impairment. Table 2 demonstrates the mean of psychosocial variables according to gender status. There were significant differences between men and women with regards to cognition status, depression symptom, and social support. The mean of cognitive impairment and depressive symptom in women were significantly higher than in men. The mean of total social support and 2 subscales (social interaction and social satisfaction) in women were significantly lower than in men. Table 3 demonstrates the univariate relationship between cognition status and psychosocial factors. There was a significant relationship between age and cognitive impairment (P < 0.001). Univariate analysis revealed that cognitive impairment was significantly more prevalent in women than in men (P < 0.001). In univariate analysis, elder people with cognitive impairment had significantly more symptoms of depression than those without cognitive impairment (P < 0.001). Elder people who lived alone had more frequency of cognitive impairment than those who did not live alone (P < 0.001). Also, older adult smokers had more frequency of cognitive impairment compared to non-smokers (P < 0.001). Those with high perceived social support had lower cognitive impairment than those with low perceived social support (P < 0.001).

Table 4 presents the results of univariate logistic regression. According to multivariate logistic regressions, depressive symptoms had a significant positive association with cognitive impairment and were considered as important risk factors in those aged 80 to 85, 70 to 74, and 75 to 79. Social support with scores ranging from 26 to 30 and 31 to 33 were negative predictors of cognitive impairment. Also, education more than 5 years was a negative predictor of cognitive impairment (Table 5).

**Table 1:** Frequency and Apercentile of Demographic Characteristics in the Elderly (N=1612)

**Variables**	**N**	**%**
Age 60-64 65-70 ≥71	571 335 706	35.4 20.8 43.8
Education Level <9 Years ≥9 years	1476 136	91.5 8.5
Marital Status Married Single	1374 238	85.2 14.8
Living Arrangement Alone With others	107 1495	6.6 93.4
Gender Male Female	881 731	54.7 45.3

*Single population includes divorced, unmarried and widowed individuals

**Table 2:** Comparison of Means of Psychosocial Factors between Men and Women of Elderly

**Variables**	**Men** **Mean (SD)**	**women** **Mean (SD)**	**P-value**
Cognition status (MMSE score)	26.2 (3.2)	23.9 (4.3)	0.001
Depression symptom (GDS score)	3.4 (2.9)	5.9 (3.5)	0.001
Social support (DSS) Social interaction Social satisfaction Total score	10.4 (1.3) 17.7 (2.5) 28.1 (3.0)	9.9 (1.5) 16.7 (2.8) 26.6 (3.4)	0.001 0.001 0.001

**Ranges: **Mini Mental State Examination (MMSE), 0-30; Geriatric Depression Scale (GDS), 0-15; Duke Social Support Index (DSSI), social interaction (1-12) and social satisfaction total score, 11-33.

**Table 3:** Univariate Relationship of the Cognitive Impairment with Psychosocial Variables in the Elderly

**Cognitive impairment variables**	**Yes**		**No**		**p-value**
	**N**	**%**	**N**	**%**	
Gender Men women	179 330	35.2 64.8	701 402	63.6 54.9	0.000
Age 60-64 65-69 70-74 75-79 80-84 85-99	120 86 87 124 58 33	21.0 25.7 30.7 48.8 50.9 63.0	452 249 196 130 56 20	79.0 74.3 69.3 51.2 49.1 37.0	0.000
Education ≤5 years >5 years	450 59	88.4 11.6	591 512	53.6 46.4	0.000
Living Alone Yes No	109 400	21.4 78.6	128 975	11.6 88.4	0.000
Smoking Yes No	451 58	88.6 11.4	860 243	78 22	0.000
Social support 11-20 21-25 26-30 31-33	45 169 236 59	83.7 49.4 27.9 16.3	16 173 610 304	26.3 50.6 72.1 83.7	0.000
Depression Yes No	300 208	59.1 40.9	399 703	36.2 63.8	0.000

**Table 4:** Predictors of Cognition Impairment in Univariate logistic Regression in the Elderly

**Variables**	**OR**	**Confidence interval (CI) 95%**	**P-value**
Gender (Women/Men)	3.25	2.49-4.25	0.000
Age 60-64 65-69 70-74 75-79 80-84 85≤	1.00 1.30 1.67 3.59 3.90 6.40	0.95-1.79 1.21-2.31 2.61-4.94 2.57-5.93 3.56-11.53	0.104 0.002 0.000 0.000 0.000
Education(>5 years/≤5 years)	0.15	0.11-0.20	0.000
Depression(Yes/No)	2.54	2.05-3.15	0.000
Social support (Duke scores) 20≥ 21-25 26-30 31-33	1.00 0.35 0.14 0.07	0.19-0.64 0.08-0.25 0.04-0.13	0.001 0.000 0.000

**Table 5:** Predictors of Cognition Impairment in Multivariate Logistic Regression in the Elderly

**Variables**	**OR**	**Confidence interval (CI) 95%**	**P-value**
Gender (Women/Men)	3.25	2.49-4.25	0.000
Age 60-64 65-69 70-74 75-79 80-84	1.18 1.30 3.05 3.47 5.81	0.82-1.69 0.91-1.88 2.11-4.41 2.13-5.65 2.99-11.22	0.352 0.148 0.000 0.000 0.000
Education(>5 years/≤5 years)	0.19	0.14-0.27	0.000
Depression (Yes/No)	1.64	1.27-2.13	0.000
Social support (Duke scores) 21-25 26-30 31-33	0.56 0.32 0.29	0.28-1.11 0.16-0.62 0.14-0.61	0.098 0.001 0.001

## Discussion

In the present study, some psychosocial predictors of cognitive impairment in the elderly were investigated. It was concluded that 509 (31.5%) elders had cognitive impairment. The prevalence of moderate to severe dementia in different population groups is 5% in the population older than 65 years (1). The prevalence of dementia in older Malaysians was reported to be 14.3% (29). In addition, we found that women had higher means of cognitive impairment and depressive symptoms compared to men. Female gender is sometimes identiﬁed as a risk factor for dementia and other psychiatric disorders (29, 30). Along with most studies, we found that women had lower social support than men (31), whereas some studies have indicated contrasting results (32, 33). 

Our results revealed that age over 70 years had a significant positive association with cognitive impairment. Similar to our study, some reports have indicated that older age is an independent risk factor for dementia (29). During aging, prevalence of dementia increases by 0.6 to 0.8 at the age of 65 to 21% at the age of 90 (1).

Moreover, we found that education more than 5 years was a negative predictor of cognitive impairment. Previous researches also indicated that lower levels of education are associated with higher risk of cognitive impairment in older adults (34). People with higher education may have a greater cognitive reserve that delays the clinical manifestations of dementia.

In our findings, depressive symptom had a significant positive association with cognitive impairment. In accordance with our results, Saczynski et al. revealed that older adults with depression have a signiﬁcant increased risk of developing dementia compared to healthy ones (35). A study investigated whether a history of depression is associated with the increased risk of dementia. In contrast to our findings, they reported that the onset of depression before dementia was not associated with an increased risk of dementia (37).

How is depression associated with cognitive impairment? Several possible underlying mechanisms have been proposed. First, depressive symptoms merely reﬂect the presence of underlying brain diseases that lead to cortical atrophy, limbic atrophy, and white matter lesions, which are often seen in both dementia and late onset depression (38). In addition, depression and dementia have common etiological factors, such as inﬂammation, vascular changes, and vascular risk factors (39, 40). Also, low socioeconomic status, educational level, and other medical comorbidities are common risk factors (41, 42).

The obtained results indicated that social support is a negative predictor of cognitive impairment. There are some hypotheses to explain the negative relationship between cognitive impairment and social support. First, social support as stress-buffering hypothesis states that people with more functional support are protected better against the negative consequences of psychological stress (43). The greater social support is associated with enhanced quality of life (16). Second, social support also increases the ability to deal with stressors via receiving informational and emotional support. If social support improves, it results in fewer physiological and psychological symptoms of the disease (44). Third, social support may also influence people, whether they are engaged in health promoting or health damaging behaviours (45). Depression and cognitive impairment frequently occur. Faramarzi et al. showed that lack of social support is negatively associated with depression in older people (46). 

## Limitation

This study has some limitations. First, we were not able to verify the relationship between some potential risk factors and development of dementia. Second, the diagnosis of cognitive impairment was not based on the clinical interview by a psychiatrist. Third, this was a cross- sectional study, and explanation of causality in these studies is difficult. However,strong points of this study were large sample size, being a population based-study, and the use of valid and reliable instruments.

## Conclusion

The findings noted that older age and depression are risk factors for cognitive impairment. Higher education level and social support are protective factors of cognitive impairment in elderly population. Further evaluations via prospective studies and eventually an experimental study on the patients are highly recommended.
